# Stereotactic Ablative Radiotherapy for Oligometastatic Pericolonic Soft Tissue Metastases Using Daily Cone-Beam Computed Tomography-Guided Online Adaptive Radiotherapy

**DOI:** 10.7759/cureus.69937

**Published:** 2024-09-22

**Authors:** Benjamin Mou, Derek Hyde, Nathan Becker

**Affiliations:** 1 Radiation Oncology, British Columbia Cancer-Kelowna (Sindi Ahluwalia Hawkins Centre), Kelowna, CAN; 2 Medical Physics, British Columbia Cancer-Kelowna (Sindi Ahluwalia Hawkins Centre), Kelowna, CAN

**Keywords:** adaptive radiation therapy, colon cancer, cone-beam computed tomography (cbct), oligometastases, online adaptive, sabr, sbrt

## Abstract

A 74-year-old woman with pathologic T4a N1 M0 adenocarcinoma of the cecum, initially treated with right hemicolectomy, developed rising serum carcinoembryonic antigen levels while receiving adjuvant chemotherapy. Re-staging investigations demonstrated two soft tissue metastases in the right abdomen comprised of a retrocolic lesion immediately posterior to the colon and a retroperitoneal lesion with no other sites of metastases. The patient was treated with stereotactic ablative radiotherapy (SABR) to a dose of 40 Gy in five daily fractions to both pericolonic soft tissue metastases simultaneously. A standard volumetric modulated arc therapy (VMAT) plan had suboptimal dose coverage of the retrocolic metastasis adjacent to the colon, so cone-beam computed tomography (CBCT)-guided online adaptive radiotherapy (ART) was employed to maximize radiation dose to the tumors due to the radioresistant histology. An intensity-modulated radiotherapy (IMRT) plan was created using artificial intelligence tools integrated with the treatment unit. Median contouring and plan creation for each fraction was 21.5 minutes (range 14.9-28.1). For the retrocolic metastasis, compared to the standard VMAT plan, the CBCT-guided online ART plan improved coverage of the gross target volume by the prescription dose from 80.0% to 99.7%. SABR to pericolonic soft tissue metastases was feasible using CBCT-guided online ART and can significantly improve target volume coverage when targets are adjacent to mobile normal organs, which may be particularly important for radioresistant histologies for local control.

## Introduction

Stereotactic ablative radiotherapy (SABR) is a treatment option for patients with oligometastatic disease. SABR delivers high precision, high-dose radiotherapy to cancer while sparing nearby organs at risk (OARs). When tumors lie in close proximity to critical OARs, a common strategy is to prioritize limiting dose to OARs over the coverage of the planning target volume (PTV), as some prospective studies demonstrate this approach is safe and does not compromise clinical outcomes [1,2]. In situations where motion and physiologic changes can alter the location of targets relative to OARs, advanced treatment approaches are often required to deliver sufficient doses to the gross tumor volume (GTV) while still meeting the constraints of OARs. This can be particularly challenging when treating radioresistant histologies that necessitate higher doses of radiation for adequate local control [3]. Online adaptive radiotherapy (ART) can account for daily variations in the shape and position of targets and OARs to optimize the delivery of radiotherapy on each day of treatment [4]. The use of artificial intelligence (AI) assisted tools can significantly speed up the daily planning time required while the patient is still on the treatment couch. This case report describes the use of daily cone-beam computed tomography (CBCT)-guided online ART to deliver SABR to a patient with oligometastatic colon cancer with two abdominal soft tissue metastases including one immediately adjacent to the colon.

## Case presentation

**Figure 1:**
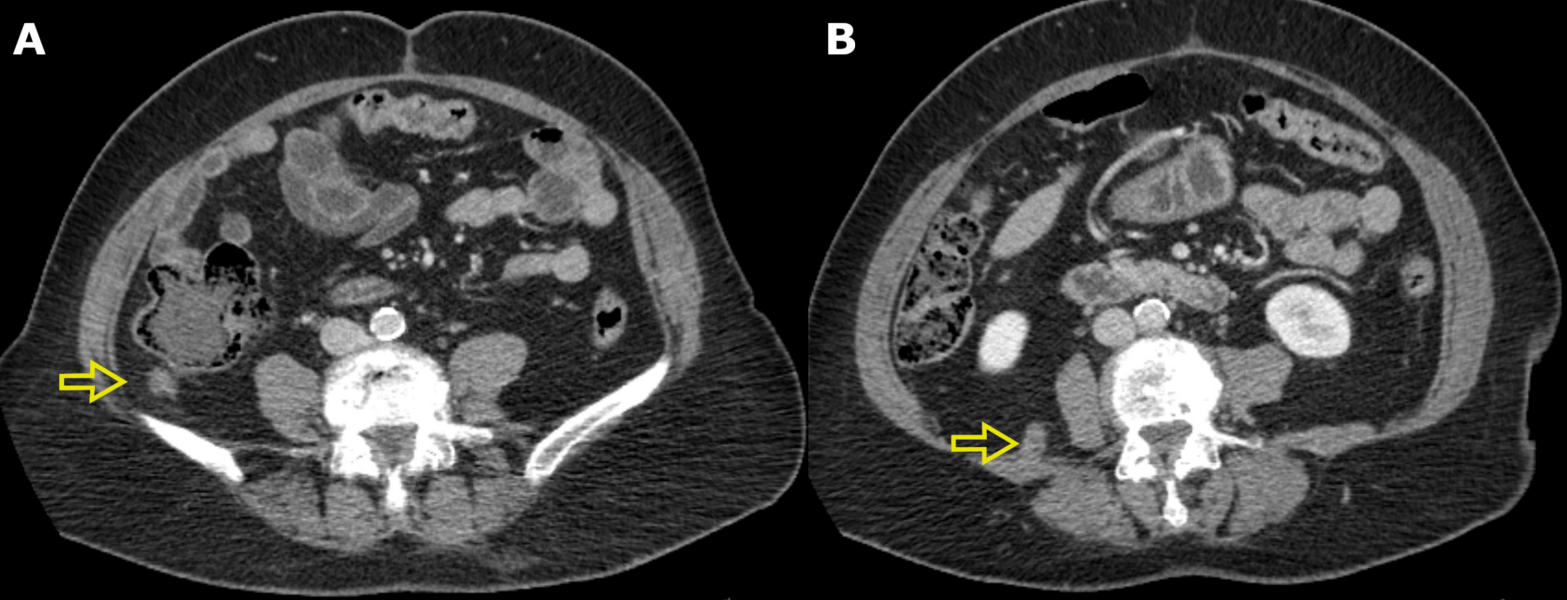
A) Retrocolic soft tissue metastasis adjacent to the ascending colon (yellow arrow). B) Retroperitoneal soft tissue metastasis in the posterior right lower quadrant (yellow arrow)

A 74-year-old woman was initially diagnosed with adenocarcinoma of the cecum and treated with a laparoscopic right hemicolectomy. The pathology demonstrated a 6.0 cm moderately differentiated adenocarcinoma invading the visceral peritoneum with one of 11 involved lymph nodes and negative margins, with a final pathologic stage of T4a N1a M0. The patient received seven out of a planned eight cycles of adjuvant chemotherapy using capecitabine and oxaliplatin. Prior to cycle eight, she had a rising serum carcinoembryonic antigen level, and re-staging computed tomography (CT) scans demonstrated progressive enlargement of two soft tissue lesions in the abdomen, measuring 13 mm and 17 mm, consistent with metastases (Figure 1). There was no evidence of other sites of metastatic disease. She was referred for consideration of SABR for the two sites of oligometastatic disease. She was asymptomatic and physical examination was unremarkable. Eastern Cooperative Oncology Group's performance status was 0. She was offered SABR for both metastases using a single isocenter due to their close proximity to each other.

**Figure 2:**
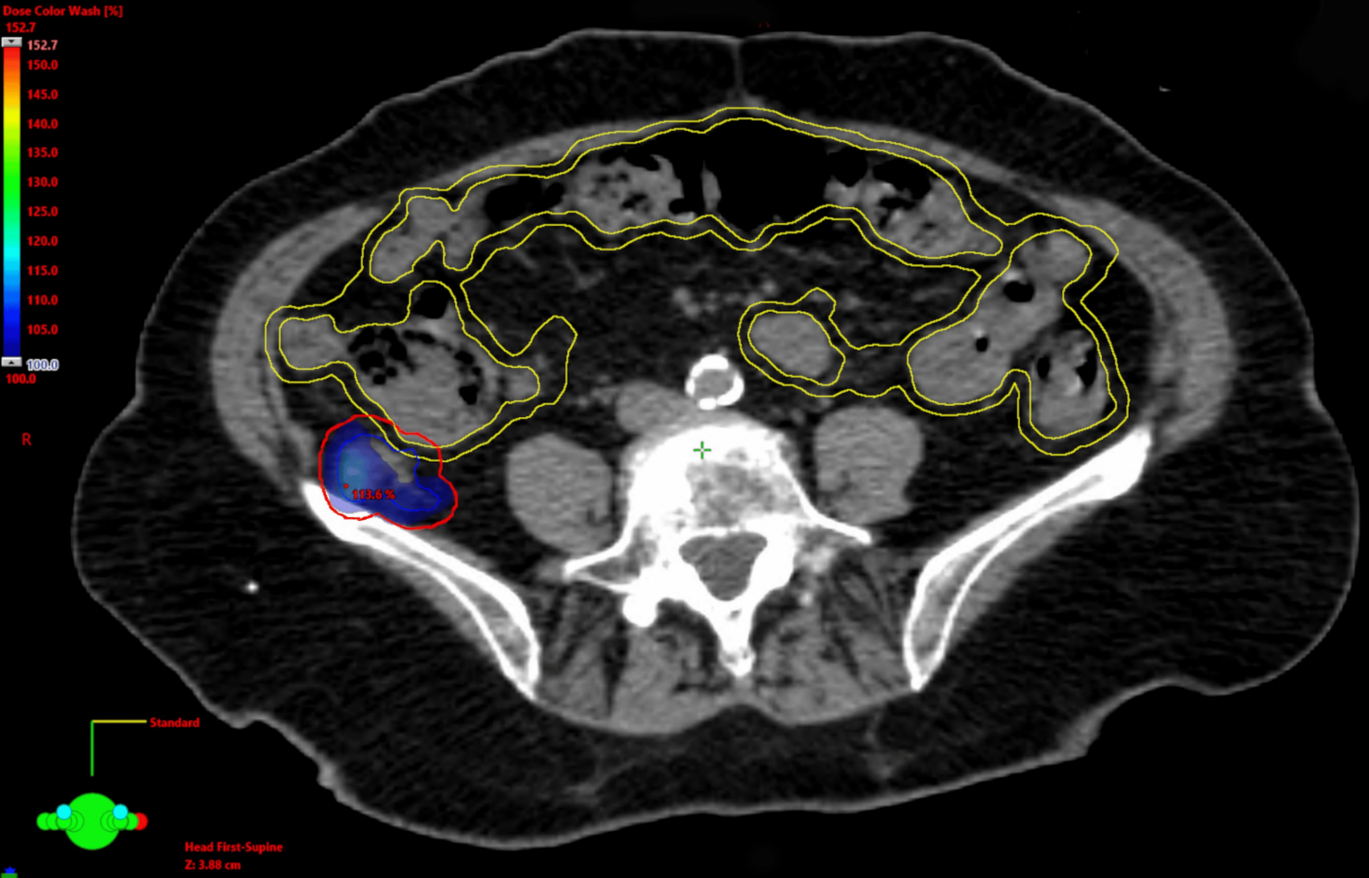
. The standard VMAT plan with color wash demonstrates under-coverage of the retrocolic GTV (blue) and PTV (red) by the prescription dose due to the proximity of the colon (yellow) VMAT: volumetric modulated arc therapy; GTV: gross tumor volume; PTV: planning target volume

A standard volumetric modulated arc therapy (VMAT) plan was created (Figure 2). The GTVs included visible disease on CT. No elective clinical target volume was used. An isometric 5 mm expansion on the GTVs was used to create the PTVs. The PTVs were both prescribed a dose of 40 Gy in five fractions delivered daily on consecutive business days. The clinical goal for GTV coverage was V100%>99.9%. The clinical goals for PTV coverage were V95%>98% and V90%>99%. No particular bowel protocol was used for this treatment site in the lower gastrointestinal tract. Given the proximity of one of the retrocolic GTVs to the colon, a 3 mm isotropic planning organ at risk volume (PRV) was applied to the colon for safety to account for daily variations in positioning relative to the GTV. The colon PRV was limited to a maximum point dose of 0.035 cc of 38 Gy. The dosimetry of this standard VMAT plan for the retrocolic metastasis demonstrated a GTV V100% of 80.0% and a PTV V95% of 81.1%. This plan was not felt to be clinically acceptable. Considering the radioresistant histology of colon cancer and the presence of two metastases in close proximity with one immediately adjacent to the right colon, the treatment team recommended using CBCT-guided online ART to create daily plans that could potentially maximize dose to the retrocolic GTV on days where there was increased space between the GTV and colon.

**Figure 3:**
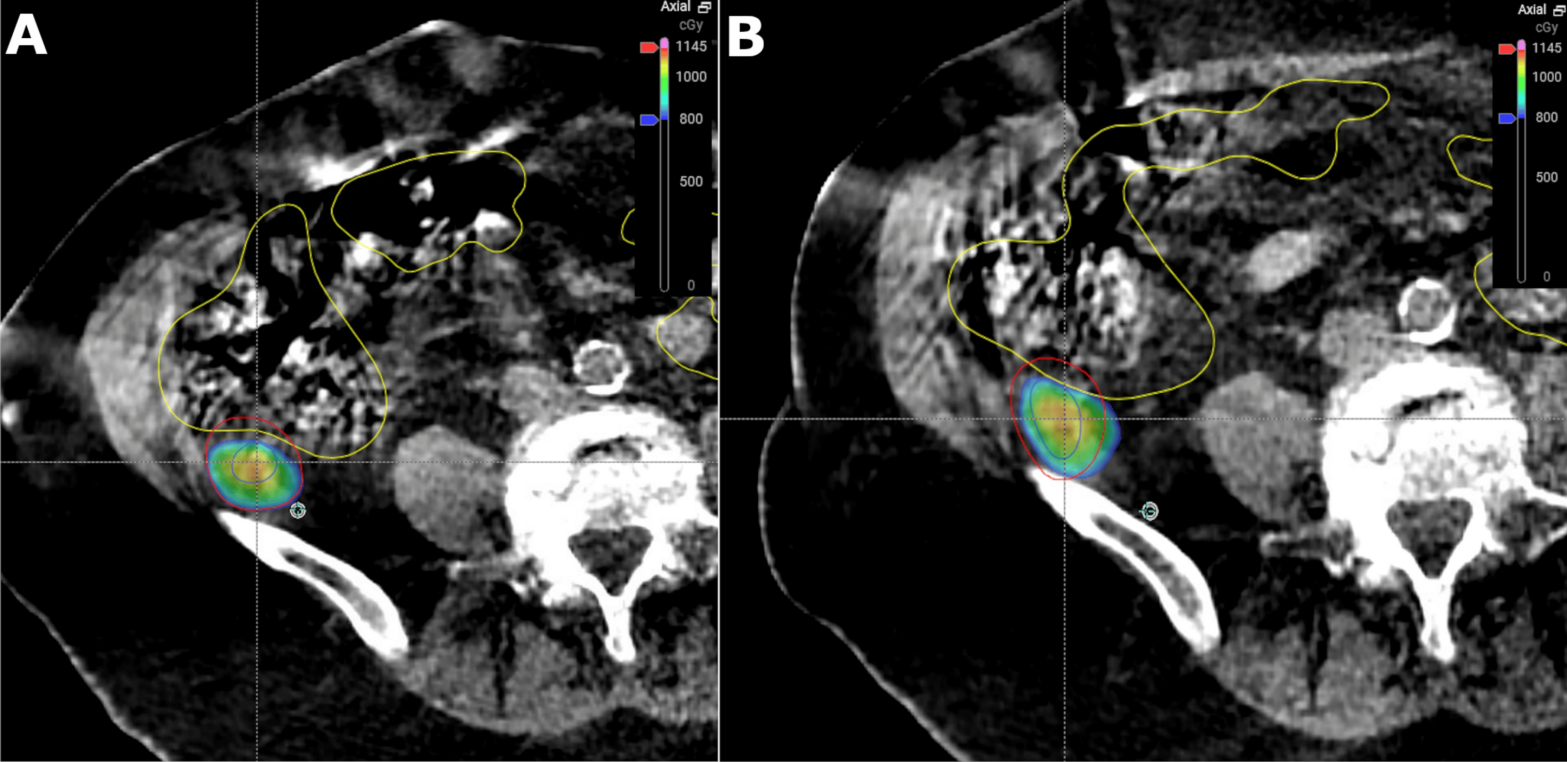
CBCT-based online adaptive plan-of-the-day for the (A) first fraction and (B) fourth fraction, with a color wash demonstrating differences in coverage of the retrocolic GTV (blue) and PTV (red) by the prescription dose, due to variations in the proximity of the colon (yellow) CBCT: cone-beam computed tomography; GTV: gross tumor volume; PTV: planning target volume

The patient was treated on the Ethos adaptive radiotherapy system (Varian, a Siemens Healthineers Company, Palo Alto, USA). Surface-guided intra-fraction monitoring was not yet implemented at the time of this patient’s treatment. A pre-plan was created using intensity-modulated radiotherapy (IMRT) techniques with nine coplanar beams. CBCT was taken prior to each fraction. A PRV on the colon was not used given that images were acquired the same day of treatment. Ethos AI tools propagated contours for the GTVs and colon. A radiation oncologist edited the AI-generated contours using the online Ethos platform focusing on structures closest to the GTVs. A 5 mm margin was applied to the modified GTV contours to create the PTV. Re-planning was performed and the plan was reviewed and approved by a radiation oncologist and medical physicist attending the treatment unit. The median time per fraction for contouring, plan generation, plan review, and approval, based on the time between the planning CBCT and treatment CBCT was 21.5 minutes (range 14.9-28.1). Table 1 summarizes the planning goals and achieved values for the target volumes and OARs in the reference plan as well as the scheduled plan and adapted plan for each fraction. Cumulative dosimetry from the online ART treatment demonstrated that 99.7% of the retrocolic GTV was covered by the prescription dose and the PTV V95% was 82.4%. The accumulated maximum dose to the colon was 38.3 Gy. The retrocolic GTV was closest to the colon for the first fraction and farthest from the colon for the fourth fraction (Figure 3). For the retroperitoneal lesion, the GTV V100% was 100% and the PTV V95% was 86.8%. There was no obvious change in the size of the GTVs over the course of treatment. There were also no acute toxicities during treatment.

**Table 1:** Planning goals and achieved metrics in the reference plan, and the scheduled and adapted plans for each fraction GTV: gross tumor volume; PTV: planning target volume; GTV1: retroperitoneal gross tumor volume; GTV2: retrocolic gross tumor volume

Structure	Planning goals	Reference plan	Fraction 1		Fraction 2		Fraction 3		Fraction 4		Fraction 5	
			Scheduled	Adapted	Scheduled	Adapted	Scheduled	Adapted	Scheduled	Adapted	Scheduled	Adapted
GTV1	V99%>99.9%	100%	100%	100%	100%	100%	100%	100%	100%	100%	100%	100%
	V125%>90%	80.9%	93.3%	78.4%	91.5%	72.7%	92.1%	77.1%	93.4%	86.3%	90.0%	79.3%
GTV2	V99%>99.9%	99.9%	99.9%	97.5%	100%	94.4%	100%	97.3%	99.6%	95.0%	97.4%	90.7%
	V125%>90%	87.3%	90.9%	76.5%	97.9%	67.4%	92.7%	76.3%	87.4%	75.4%	83.2%	60.0%
PTV1	V90%>99.5%	100%	100%	98.9%	99.9%	96.4%	99.9%	96.4%	100%	99.6%	99.9%	98.9%
	V95%>98%	99.7%	99.9%	94.9%	99.8%	90.0%	99.8%	90.0%	99.9%	97.3%	99.8%	95.4%
	D0.04cc <140%	130.4%	132.6%	134.6%	133.2%	137.1%	132%	135.7%	133.0%	135.7%	132.8%	136.1%
PTV2	V90%>99.5%	96.4%	98.4%	87.9%	99.7%	82.3%	98.4%	84.9%	97.3%	85.4%	90.6%	81.6%
	V95%>98%	94%	96.4%	80.3%	99.0%	74%	96.6%	76.3%	93.9%	79.6%	88.0%	74.8%
	D0.04cc <140%	135.7%	136.8%	144.8%	137.2%	147.3%	137.8%	142.9%	138.3%	143.7%	137.8%	142.3%
Bowel	D0.04cc <740 cGy	735 cGy	1059 cGy	746 cGy	1059 cGy	760 cGy	1080 cGy	739 cGy	1008 cGy	757 cGy	1094 cGy	755 cGy
	V500cGy <15 cc	4.18 cc	14.22 cc	6.97 cc	15.84 cc	6.87 cc	13.85 cc	5.81 cc	12.66 cc	8.38 cc	19.49 cc	10.78 cc
Kidney-right	D0.04cc <640 cGy	336 cGy	330 cGy	355 cGy	355 cGy	387 cGy	261 cGy	307 cGy	305 cGy	376 cGy	274 cGy	299 cGy

At nine months post-SABR, the two abdominal soft tissue metastases were locally controlled. The patient remained completely asymptomatic and was able to avoid restarting chemotherapy during this time.

## Discussion

To the best of our knowledge, this case report describes the first use of CBCT-guided online ART to deliver SABR to pericolonic soft tissue metastases in a patient with oligometastatic colon cancer. Compared to a standard VMAT plan, the CBCT-guided online ART plan improved the retrocolic GTV V100% from 80.0% to 99.7%. The radioresistant nature of colon cancer requires ablative doses of radiotherapy to provide adequate local control [3,5,6]. The retrocolic GTV in this case would have been significantly under-covered by the prescription dose because the target was adjacent to the normal colon. Using traditional CT simulation-based planning techniques, our institution would typically add a PRV to the colon to account for uncertainties and variations in positioning between the time of CT simulation and the daily treatments typically scheduled two weeks later. However, the use of a PRV would further reduce the coverage of the GTV and PTV, which could put the patient at higher risk of local failure. By using online ART, the exact location of the colon relative to the GTV could be taken into account in order to limit the dose to the GTV when it abutted the colon on some days while increasing the dose to the GTV on days when there was increased distance between the GTV and colon (Figure 3). This adaptive approach allows for personalized, precision treatment that balances the need to respect OAR dose tolerances while increasing the dose to the GTV when it is safe to do so and consequently avoiding the use of a PRV. Another benefit of online ART is the ability to treat multiple targets that may move independently of one another in the same treatment session. The dose around each target can be individually sculpted based on individual target and OAR requirements so that compromise coverage to one target may not be required based on the movement of another, as is often required with standard image-guided radiotherapy. Although the follow-up time is short, SABR was able to prevent further radiologic growth of the metastases while keeping the patient asymptomatic and avoiding the need to restart chemotherapy, which can have a tangible impact on a patient’s quality of life [7,8]. The ability of online ART to improve the therapeutic ratio in such challenging situations may allow for complex cases to be treated effectively with superior dosimetry than non-adaptive methods. Further studies are needed to assess if online ART can lead to reductions in PTV margins, which has been investigated for other treatment sites [9-11].

The use of online ART is more resource-intensive, as it requires additional personnel at the treatment unit to modify and approve the plan while the patient is on the treatment couch. SABR cases are well-suited for online ART because they typically only involve one to five fractions. More commonly treated sites requiring several weeks of fractionated radiotherapy may benefit from workflows where other team members, such as advanced practice radiation therapists, may be trained to complete the contouring functions, which will improve overall workflow times and reduce physician workload [12]. Alternatively, a schedule of radiation oncologists covering online ART cases may be an option for larger centers with sufficient radiation oncologists trained in online ART. In contrast to magnetic resonance image-guided online ART, which typically has longer image acquisition and planning times, CBCT-guided online ART fractions can be scheduled in a 40-minute treatment slot [13]. The ability of online ART platforms to function as standard linear accelerators capable of delivering non-adaptive treatments significantly improves the efficiency of the machine for general use in busy clinics. The clinical advantages of online ART must be balanced with the increased human resource requirements and associated opportunity costs [14]. Optimizing the workflow is an ongoing process and new technological features with upgraded software may facilitate the adoption of online ART in a wider range of centers.

## Conclusions

SABR to pericolonic soft tissue metastases was feasible using daily CBCT-guided online ART techniques. This approach maximized radiation dose to the GTV when it was close to the colon by accounting for the position of targets relative to OARs on the day of treatment. This technique is particularly useful for treating radioresistant histologies that require maximal ablative doses to improve the chance of local control. Further research into the use of CBCT-guided online ART for SABR in oligometastatic radioresistant tumors is warranted.
